# Prefrontal GABA levels, hippocampal resting perfusion and the risk of psychosis

**DOI:** 10.1038/s41386-017-0004-6

**Published:** 2018-01-30

**Authors:** Gemma Modinos, Fatma Şimşek, Matilda Azis, Matthijs Bossong, Ilaria Bonoldi, Carly Samson, Beverly Quinn, Jesus Perez, Matthew R Broome, Fernando Zelaya, David J Lythgoe, Oliver D Howes, James M Stone, Anthony A Grace, Paul Allen, Philip McGuire

**Affiliations:** 1 Department of Psychosis Studies, Institute of Psychiatry, Psychology & Neuroscience, King’s College London, London, UK; 2 Department of Neuroimaging, Institute of Psychiatry, Psychology & Neuroscience, King’s College London, London, UK; 3 Department of Psychiatry, Brain Center Rudolf Magnus, University Medical Center Utrecht, Utrecht, Netherlands; 4 CAMEO Early Intervention in Psychosis Service, Cambridgeshire and Peterborough NHS Foundation Trust, Cambridge, UK; 5 Department of Psychiatry, University of Cambridge, Cambridge, UK; 6 Department of Neuroscience, Instituto de Investigacion Biomedica de Salamanca (IBSAL), University of Salamanca, Salamanca, Spain; 7 Department of Psychiatry, University of Oxford, Oxford, UK; 8 Oxford Health NHS Foundation Trust, Oxford, UK; 9 Department of Neuroscience, Psychiatry and Psychology, University of Pittsburgh, Pittsburgh, PA USA; 10 Department of Psychology, University of Roehampton, Roehampton, UK

## Abstract

Preclinical models propose that the onset of psychosis is associated with hippocampal hyperactivity, thought to be driven by cortical GABAergic interneuron dysfunction and disinhibition of pyramidal neurons. Recent neuroimaging studies suggest that resting hippocampal perfusion is increased in subjects at ultra-high risk (UHR) for psychosis, but how this may be related to GABA concentrations is unknown. The present study used a multimodal neuroimaging approach to address this issue in UHR subjects. Proton magnetic resonance spectroscopy and pulsed-continuous arterial spin labeling imaging were acquired to investigate the relationship between medial prefrontal (MPFC) GABA+ levels (including some contribution from macromolecules) and hippocampal regional cerebral blood flow (rCBF) in 36 individuals at UHR of psychosis, based on preclinical evidence that MPFC dysfunction is involved in hippocampal hyperactivity. The subjects were then clinically monitored for 2 years: during this period, 7 developed a psychotic disorder and 29 did not. At baseline, MPFC GABA+ levels were positively correlated with rCBF in the left hippocampus (region of interest analysis, *p* = 0.044 family-wise error corrected, FWE). This correlation in the left hippocampus was significantly different in UHR subjects who went on to develop psychosis relative to those who did not (*p* = 0.022 FWE), suggesting the absence of a correlation in the latter subgroup. These findings provide the first human evidence that MPFC GABA+ concentrations are related to resting hippocampal perfusion in the UHR state, and offer some support for a link between GABA levels and hippocampal function in the development of psychosis.

## Introduction

Post-mortem and preclinical studies have provided consistent evidence that the pathophysiology of psychotic disorders involves an alteration in GABAergic neurotransmission [1, 2]. More specifically, schizophrenia has been linked to a defect in glutamate decarboxylase (GAD67) mRNA in parvalbumin-expressing (PV+) interneurons within a corticolimbic circuit involving the prefrontal cortex and the hippocampus [3, 4]. Recent work on a neurodevelopmental animal model of psychosis [5] indicates that medial prefrontal cortex (MPFC) dysfunction leads to increased stress-induced functional loss of hippocampal PV+ interneurons [6], which is associated with hippocampal hyperactivity through disinhibition of glutamatergic pyramidal cells [7]. Increased glutamatergic output from the ventral hippocampus is hypothesized to then lead to elevated subcortical dopamine function, resulting in some of the behavioral abnormalities associated with schizophrenia [8]. Furthermore, independent research using other preclinical models shows that mutations in genes implicated in schizophrenia, such as ERBB4 and dysbindin, are associated with disrupted PV+ interneuron function and dysregulation of pyramidal cell activity [9, 10].

Data from post-mortem and preclinical studies thus suggest that cortical GABAergic function is reduced in schizophrenia, and that this can be detected in the premorbid stages of the disorder [11]. GABA concentrations can be quantified in vivo using proton magnetic resonance spectroscopy (^1^H-MRS). Nonetheless, studies comparing MPFC GABA levels in patients and controls have reported inconsistent findings, including decreases [12–14], increases [15], and no significant differences [16–19]. Indeed, a recent meta-analysis did not find a significant difference in regional GABA levels between patients with schizophrenia and healthy volunteers [20]. There have been relatively few ^1^H-MRS GABA studies in subjects at ultra-high risk (UHR) of developing psychosis, and all of these have examined GABA in the MPFC. One study reported higher levels in subjects at UHR compared with controls [21]; one described lower GABA levels in UHR subjects than in controls [22], and two studies including a recent one from our group found no differences between UHR subjects and healthy controls [23, 24]. Despite these inconsistencies, while preclinical models suggest that MPFC dysfunction leads to hippocampal overdrive in psychosis [6], this has yet to be explicitly investigated in humans.

Regional cerebral blood flow (rCBF) is directly correlated with the level of local neural activity [25] and can be quantitatively measured using arterial spin labeling (ASL), which uses magnetically labeled blood as endogenous tracer [26]. Studies using ASL suggest that resting perfusion is increased in the hippocampus in individuals at UHR of psychosis [27, 28], and similar findings in UHR have been reported using other magnetic resonance imaging methods involving intravenous injections of the contrast agent gadolinium to measure basal cerebral blood volume [29]. In patients with schizophrenia compared to healthy controls, hyperperfusion in the hippocampus has been documented [30–33], as well as in other brain regions such as the basal ganglia and middle temporal lobes [34], cerebellum, brainstem and thalamus [35], although the latter two studies did not report significant effects in the hippocampus. The aim of the present study was to investigate whether resting perfusion of the hippocampus in UHR individuals may be related to GABA levels in the prefrontal cortex. We used pulsed-continuous arterial spin labeling (pCASL) to measure resting hippocampal perfusion and ^1^H-MRS to examine MPFC GABA levels in a sample of individuals at UHR of developing psychosis. For ^1^H-MRS, the MPFC was chosen as (i) this is the most widely researched region in ^1^H-MRS GABA studies of schizophrenia/UHR subjects [36], (ii) preclinical models indicate that MPFC deficits dysregulate hippocampal activity [6], and (iii) measurement of hippocampal GABA with ^1^H-MRS is highly technically challenging. We tested the hypothesis that MPFC GABA levels would be negatively associated with hippocampal perfusion in UHR subjects. A further prediction was that this association would be strongest in the subgroup of UHR subjects who subsequently developed psychosis.

## Materials and methods

### Participants

Ethical approval for the study was obtained from the Research Ethics Committee of King’s College London and South London and Maudsley (SLaM) NHS Trust, and all participants provided informed consent. Males and females aged 18–30 were invited to participate and the study was completed by 36 subjects at UHR of psychosis.

Participants were recruited from three different clinical sites, but they all underwent pCASL and ^1^H-MRS scanning on a General Electric Signa HDx TwinSpeed 3T scanner (Milwakee, Wisconsin) at the Centre for Neuroimaging Sciences, Institute of Psychiatry, Psychology & Neuroscience (King’s College London), in a single session. All clinical assessments were conducted on the same day of scanning at King’s College London by trained researchers. The different sites were: OASIS (Outreach and Support in South London) [37], part of the SLaM NHS Trust (*n* = 22); CAMEO, part of the Cambridge and Peterborough NHS Trust (*n* = 11); the West London Early Intervention Service (*n* = 2); and the Coventry and Warwick NHS Trust (*n* = 1). Inclusion criteria involved the presence of one (or more) of the following: (1) attenuated psychotic symptoms (APS), (2) a brief psychotic episode of less than 1 week’s duration that spontaneously remits without antipsychotic medication or hospitalization (Brief Limited Intermittent Psychotic episode), and (3) trait vulnerability (schizotypal personality disorder or a first-degree relative with psychosis) plus a marked decline in psychosocial functioning (Global Assessment of Functioning, GAF) [38]. UHR signs and symptoms for inclusion criteria were assessed with the Comprehensive Assessment of At-Risk Mental States (CAARMS) [39], a semi-structured interview designed to assess prodromal psychopathology in people at UHR for psychosis. All UHR subjects were antipsychotic-naïve and none were on benzodiazepines at the time of scanning. Eleven out of the 36 participants were currently taking antidepressant medications. Exclusion criteria were past/present diagnosis of psychotic disorders, past/present/familiar history of neurological illness, substance abuse/dependence as defined using DSM-5 criteria [38], or contraindication to scanning. All subjects had an estimated premorbid IQ in the normal range as assessed with the Wechsler Adult Intelligence Scale-III (WAIS-III) [40].

### Clinical measures

At the time of the scan, the following measures were collected: prodromal symptomatology using the CAARMS [39]; anxiety and depression symptoms using the Hamilton Anxiety and Depression Scales (HAM-A / HAM-D) [41]; and social and occupational functioning using the GAF [38]. Medication history and use of alcohol, tobacco, and illicit drugs was assessed using a semi-structured interview adapted from the Early Psychosis Prevention and Intervention Centre (EPPIC) Drug and Alcohol Assessment Schedule (http://www.eppic.org.au). At follow-up, clinical outcomes were determined using the CAARMS Psychosis Threshold criteria [39] and confirmed with the Structured Clinical Interview for Diagnosis [38] as administered by an experienced psychiatrist. Seven of the UHR subjects (19%) developed a psychotic disorder (the psychotic transition group) within the follow-up period (18 months). These disorders comprised: schizophrenia (*n* = 3), schizoaffective disorder (*n* = 1), and bipolar disorder (*n* = 3).

### pCASL acquisition and preprocessing

Parameters for data acquisition, computation of CBF maps, and procedures for spatial normalization of these maps to the reference space of the Montreal Neurological Institute (MNI) were identical to those described in a separate, non-overlapping UHR sample [28].

In brief, four pairs of control-labeled pCASL images were acquired using a 3D Fast Spin Echo (FSE) stack-of-spiral multi-shot readout, after a post-labeling delay of 1.5 s. Labeling of arterial blood was achieved using a flow-driven adiabatic inversion approach [42], consisting of a train of 1000 Hanning-shaped RF pulses with a duration of 500 s and an inter-pulse gap of 1ms. Parameters of the image readout were as follows: TE = 32.25 ms; TR = 5500 ms; field of view = 240; flip angle = 90°; 60 contiguous slice location of thickness 3 mm were obtained to achieve whole-brain coverage. To maximize sensitivity to blood perfusion, background suppression was achieved by selective saturation of the image slab at 4.3 s before acquisition, selective inversion 3 s before acquisition, and non-selective inversions at 1.5 s, 764 ms, 334 ms, and 84 ms before imaging. A proton-density calibration image was collected with the same sequence. This image was used to quantify blood flow in physiological units (ml blood/100 g tissue/min) following the guidelines recently published for the computation of CBF [43]. The complete ASL pulse sequence including the proton-density image was performed in 6 min. For image registration a high-resolution T2-weighted Fast-Relaxation Fast Spin Echo (FR-FSE) image (TE = 65.28 ms, TR = 4380 ms, flip angle = 90°, FoV = 240, slice thickness = 2 mm, matrix = 320 × 320 mm) was employed.

CBF maps were preprocessed using FMRIB Software Library (FSL) software applications (http://www.fmrib.ox.a.c.uk/fsl) and Statistical Parametric Mapping (SPM8; http://www.fil.ion.ucl.ac.uk/spm/). A multi-step approach was performed including the pCASL, the T2, and the SPGR scans: (1) elimination of extra-cerebral signal from the T2 scan using the “Brain Extraction Tool” (BET) of FSL7, and co-registration of the skull-stripped T2 volume and its corresponding T2 binary mask to the pCASL scan; (2) multiplication of the pCASL scan (rCBF map) with the co-registered brain binary mask to remove extra-cerebral signal from this scan; (3) co-registration back to the original T2 scan of the skull-stripped CBF map following step (2); (4) normalization of the subject’s T2 and multiplied pCASL (step 2) with the T2 template from SPM. Finally, spatial smoothing of the normalized individual CBF maps was carried out using a 6 mm Gaussian smoothing kernel.

### ^1^H-MRS acquisition and quantification

**Fig. 1:**
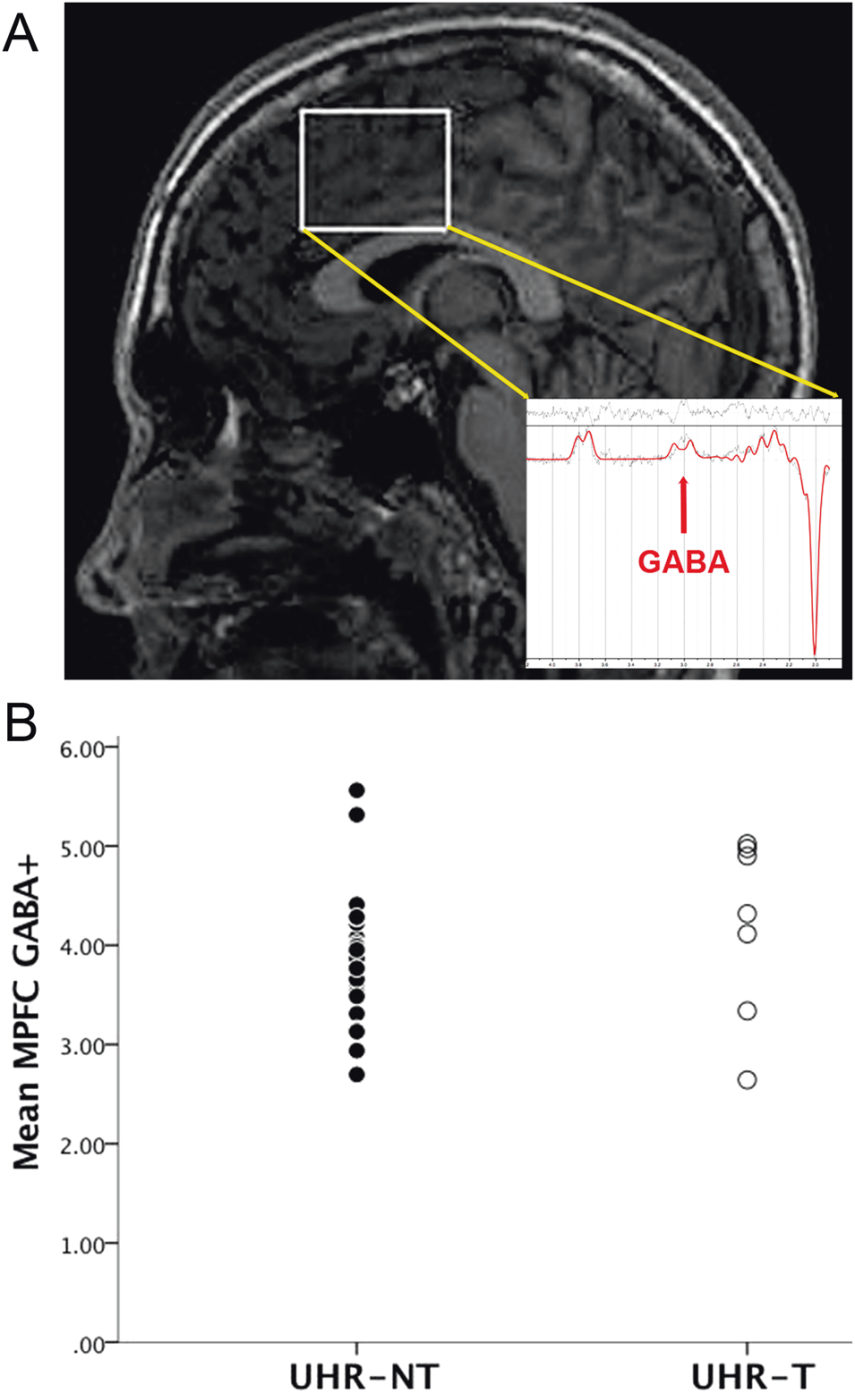
**a** Baseline GABA+ levels were not directly linked to transition outcomes (*n*=7). Location of MEGA-PRESS voxel on the medial prefrontal cortex and representative MRS spectrum. **b** Mean GABA+ concentrations by group. Light bars represent ultra-high-risk subjects who later transitioned to psychosis and dark bars those who did not

GABA+ levels (including some signal from unrelated macromolecules, i.e. diverse proteins and lipids) were obtained from the MPFC using MEGA-PRESS, which incorporates a standardized chemically selective suppression (CHESS) water suppression routine (TE = 68ms, TR = 2000ms, 320 averages). For each acquisition, unsuppressed water reference spectra (16 averages) were also acquired. Shimming was optimized, with auto-prescan performed twice before each scan. The region of interest (ROI) in the MPFC was prescribed from the midline sagittal localizer, and the center of the 40 × 25 × 30 mm ROI, mostly covering the MPFC but also including some contribution from anterior cingulate cortex, was placed above the middle section of the corpus callosum (Fig. 1a). MEGA-PRESS scan duration was ~13 min. Structural data were acquired by means of a three-dimensional T1-weighted magnetization prepared rapid acquisition gradient-echo sequence (TR = 6.98 ms, TE = 2.85 ms, voxel size = 1.05 × 1.05 × 1.2 mm, FoV = 260 mm, flip angle = 11°, inversion time = 400 ms).

Spectra were analyzed using LCModel version 6.3-1L (http://s-provencher.com/pages/lcmodel.shtml) with the basis set provided by its author [44, 45]. Water-scaled GABA+ values were corrected for the voxel tissue composition by using the formula: Metabolite Corrected = Metabolite Concentration × [proportion white matter + (1.283 × proportion gray matter) + (1.55 × proportion corticospinal fluid)]/(proportion white matter + proportion gray matter) [46]. Voxel gray matter (GM), white matter (WM), and corticospinal fluid (CSF) content for each subject were determined by extracting the location of the voxel from the spectra file headers, and using an in-house program to calculate the percentage of GM, WM, and CSF content using the segmented T1-weighted images. We used (i) Cramer-Rao minimum variance bounds (CRLB) > 20% as reported by LCModel, which are estimates of fit of the metabolite peaks, and (ii) signal-to-noise ratio (SNR) < 8 to exclude poorly fitted metabolite peaks from statistical analysis [45, 47]. Data from all 36 participants in the present study met these criteria. The GABA ^1^H-MRS data from the male participants in this sample (*n* = 21) overlap with the dataset recently reported as part of a case–control study in males [23]. The primary ^1^H-MRS measure was GABA+ normalized to water concentrations are given in “institutional units”.

### Statistical analysis

#### Demographic data

Analysis of demographic data was performed with the Statistical Package for Social Sciences (SPSS) version 24 (Chicago, IL). After confirming homogeneity of variance with Levene’s test, the effect of group (psychotic transition, non-transition) on demographic and clinical variables was tested using independent samples *t-*tests for parametric data and Chi-square tests for non-parametric data. Significant effects are reported at *p* < 0.05, two tailed.

#### ^1^H-MRS analysis

Between-group differences in MPFC GABA+ concentrations were examined with an independent samples *t*-test in SPSS. Levene’s test was used to check for equality of variance across groups.

#### pCASL analysis

Between-group differences in rCBF were examined with an independent samples *t*-test using Statistical Parametric Mapping Version 8 (http://www.fil.ion.ucl.ac.uk/spm/software/spm8), including age and gender as covariates of no interests. Hippocampal ROIs were specified a priori using the coordinates from a previous rCBF study in a separate UHR sample [28]: MNI coordinates right hippocampus *x*, *y*, *z* = 20, −28, −8 and left hippocampus *x*, *y*, *z* = −22, −28, −8. These coordinates were used for small volume correction with a 10 mm sphere. Results were considered significant after *p* < 0.05 with family-wise error correction (FWE). For completeness, exploratory whole-brain analyses were performed and are reported when surviving *p* < 0.05 FWE correction. This second level model used the global average CBF value over the GM volume of each subject as a covariate, in order to account for inter-individual differences in global perfusion [48].

#### Integration of pCASL and ^1^H-MRS data

The relationship between hippocampal rCBF and MPFC GABA+ levels was also investigated in SPM8. Individual GABA+ values were entered as regressors in a voxel-wise ANOVA, using age and gender as covariates of no interest, to examine (i) rCBF-GABA+ associations across the UHR sample, as well as (ii) group differences in rCBF-GABA+ associations in UHR subjects who later transitioned to psychosis (UHR-T) compared with those who did not (UHR-NT). Hippocampal ROIs were specified a priori using the same coordinates as described above, and results were considered significant after *p* < 0.05 FWE. For completeness, exploratory whole-brain analyses were performed and are reported when surviving *p* < 0.05 FWE correction. As above, the global average CBF value over the GM volume of each subject was used as a covariate to account for inter-individual differences in global perfusion [48].

Finally, potential effects of use of antidepressant medication or substances (tobacco, cannabis, and alcohol) on our outcome measures (GABA+ levels, rCBF, GABA+, and rCBF interactions) were assessed either in SPSS or by adding those variables as covariates in the SPM designs. Similarly, associations between GABA+ levels and prodromal symptom severity were tested using Pearson’s product-moment correlation in SPSS.

## RESULTS

Table 1 summarizes the participant’s characteristics. All subjects met the APS criteria of the UHR state. The UHR-NT and UHR-T subgroups were not significantly different in terms of age (*p* = 0.734), gender (*p* = 0.943), estimated IQ (*p* = 0.968), cigarette (*p* = 0.182), cannabis (*p* = 0.797), alcohol use (*p* = 0.399), or antidepressant use (*p* = 0.899).

**Table 1:** Participant demographic and clinical characteristics at presentation

	Total UHR (*n* = 36)	Non-transition (*n* = 29)	Transition (*n* = 7)	UHR-NT vs UHR-T	
	Mean (SD)	Mean (SD)	Mean (SD)	Statistic	*p*
Age (years)	21.8 (2.9)	21.7 (2.9)	22.1 (3.0)	*t* = 0.342	0.734
Gender (male/female)	21/15	17/12	4/3	*χ*^2^ = 0.005	0.943
Estimated IQ	107.6 (11.6)	107.8 (12.3)	106.2 (7.3)	*t* = −0.276	0.784
CAARMS positive	11.9 (4.2)	11.8 (4.1)	12.6 (3.3)	*t* = 0.443	0.661
CAARMS negative	6.9 (4.42)	6.8 (4.2)	7.1 (5.7)	*t* = 0.173	0.864
GAF	58.3 (11.6)	58.5 (11.3)	57.9 (13.5)	*t* = −0.121	0.905
HAM-A	21.0 (11.7)	18.6 (11.0)	28.4 (11.4)	*t* = 2.046	0.051
HAM-D	19.2 (11.3)	17.4 (11.9)	24.6 (7.6)	*t* = 1.485	0.149
Tobacco (cigarettes/day)	5.4 (8.2)	6.4 (8.8)	1.7 (3.3)	*t* = −1.362	0.182
Alcohol (units/day)	2.3 (4.6)	2.7 (5.1)	1.0 (0.6)	*t* = −0.854	0.399
Cannabis (median [range])	0 (0–4)	0 (0–4)	1 (0–4)	*χ*^2^ = 1.667	0.797
Antipsychotic medication	0	–	–	–	–
Benzodiazepines	0	–	–	–	–
Antidepressant medication (y/n)	11/25	9/20	2/5	*χ*^2^ = 0.016	0.899

*CAARMS* Comprehensive Assessment of At Risk Mental States, Cannabis/alcohol use: 0 = never, 1 = experimental use (has tried occasionally), 2 = occasional use (has used small quantities from time to time), 3 = moderate use (has used in small quantities regularly / large amounts occasionally), 4 = severe use (has frequently used large quantities, often to intoxication/debilitation), *GAF* Global Assessment of Functioning, *HAM-A/D* Hamilton Anxiety and Depression Scales

### ^1^H-MRS spectral quality

Spectra obtained were of good quality, with LCModel reporting mean ± SD signal-to-noise ratio of 21.94 ± 3.46, line width of 6.84 ± 2.92 Hz. UHR subjects who transitioned to psychosis did not differ from UHR-NT subjects in any of the parameters relating to GABA+ spectral quality or voxel tissue content (Table 2).

**Table 2:** ^1^H-MRS quality parameters and metabolite levels

	Total UHR (*n* = 36)	Non-transition (*n* = 29)	Transition (*n* = 7)	UHR-NT vs UHR-T	
	Mean (SD)	Mean (SD)	Mean (SD)	Statistic	*p*
SNR	21.9 (3.5)	21.8 (3.2)	22.7 (4.7)	0.650	0.520
Line width	6.8 (2.9)	7.0 (3.1)	6.2 (1.7)	−0.692	0.494
Voxel CSF	0.1 (0.1)	0.1 (0.0)	0.2 (0.1)	0.539	0.593
Voxel GM	0.5 (0.4)	0.5 (0.0)	0.5 (0.0)	−0.497	0.622
Voxel WM	0.3 (0.1)	0.3 (0.0)	0.3 (0.1)	−0.059	0.953
GABA+	3.9 (0.7)	3.8 (0.6)	4.2 (0.9)	1.245	0.222
GABA+ % CRLB	5.8 (1.9)	5.9 (1.5)	5.3 (1.4)	−0.948	0.350

*CRLB* Cramer-Rao Lower Bounds, *CSF* cerebrospinal fluid, *GM* gray matter, *SNR* signal-to-noise ratio, *WM* white matter

### ^1^H-MRS in UHR individuals: relationship to clinical outcome

There was no difference in GABA+ levels between the UHR-T and UHR-NT subgroups (*t* = −1.25; *p* = 0.222; Fig. 1b, Table 2). Substance use and antidepressant medication had no significant effect on GABA+ levels (tobacco: *r* = −0.094, *p* = 0.591; alcohol: *r* = −0.057, *p* = 0.746; cannabis: *F*_4,34_ = 0.484, *p* = 0.748; antidepressants: *t* = −0.005, *p = *0.996), and there was no association between GABA+ concentrations and levels of CAARMS positive (*r* = −0.027, *p* = 0.877) or negative symptoms (*r* = −0.119, *p* = 0.495).

### rCBF in UHR individuals: relationship to clinical outcome

Voxel-wise ROI analysis on hippocampal rCBF showed no significant differences between UHR-NT and UHR-T surviving *p* < 0.05 FWE correction. At the whole-brain level, UHR-T subjects showed lower rCBF than UHR-NT subjects along a cortical midline region encompassing the paracentral lobule and the supplementary motor area (*x*, *y*, *z* = 2, −32, 60; *T* = 5.51; *Z* = 4.59; *p* = 0.040 FWE) (Figure S1). These findings remained unchanged after adding tobacco, alcohol, and cannabis use as covariates of no interest in the analysis (no suprathreshold voxels in hippocampal ROI analysis, but lower rCBF in UHR-T subjects in the cortical midline region *x*, *y*, *z* = 2, −32, 60; *T* = 5.62; *Z* = 4.58; *p* = 0.044 FWE).

### GABA+ levels and hippocampal rCBF in UHR individuals

**Fig. 2:**
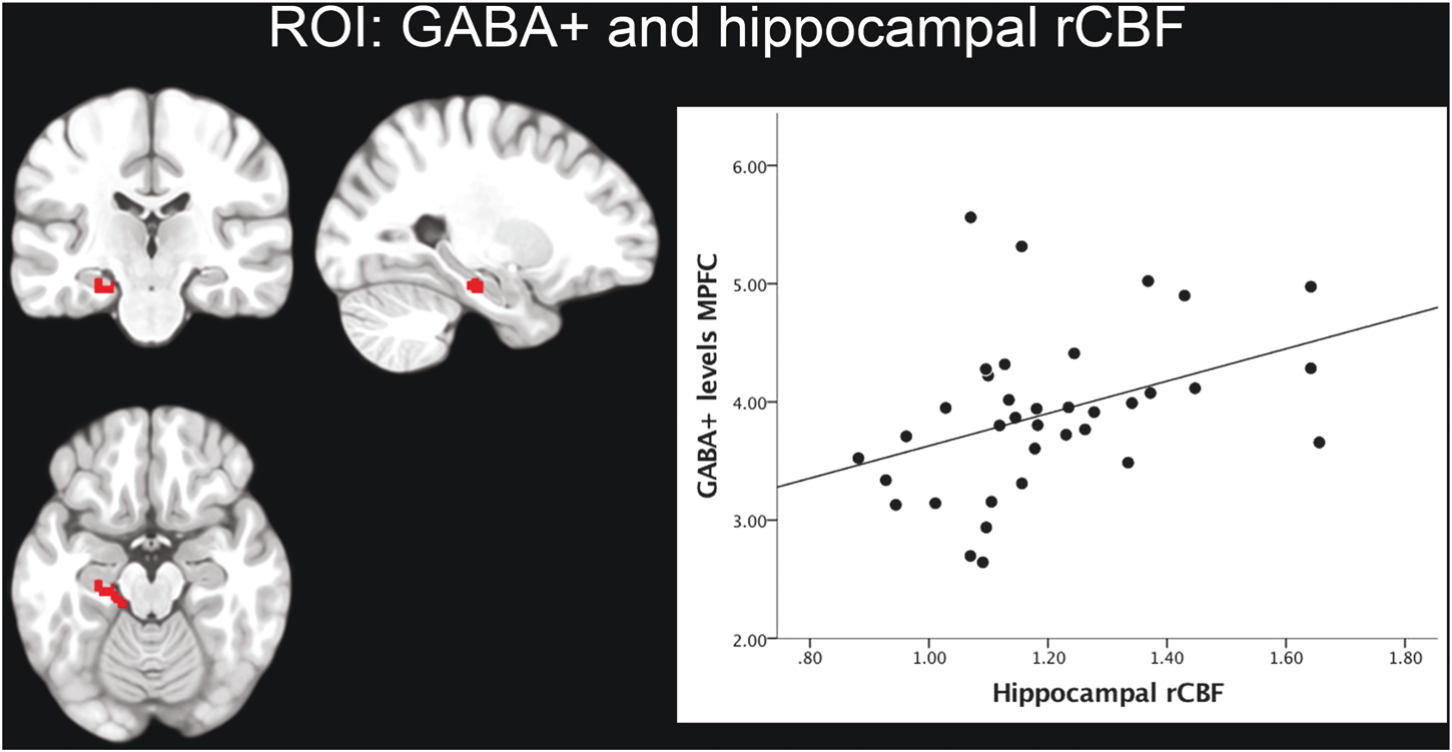
Baseline positive association in UHR subjects between levels of GABA+ in the MPFC and rCBF in the left hippocampus. rCBF values are expressed as ratio over global rCBF. Significant effects at *p* < 0.05 FWE, shown at *p* < 0.005 uncorrected for display purposes

Figure 2 shows the relationship between GABA+ levels and rCBF in the UHR group, independent of clinical outcome. ROI analysis revealed a significant *positive* association between MPFC GABA+ levels and rCBF in the left hippocampus (*x*, *y*, *z* = −26, −20, −4; *T* = 3.47; *Z* = 3.16; *p* = 0.044 FWE). This result remained significant when adding tobacco, alcohol, and cannabis use as covariates of no interest (*x*, *y*, *z* = −28, −18, −6; *T* = 3.30; *Z* = 3.00; *p* = 0.040 FWE).

Voxel-wise whole-brain analysis revealed a *negative* association between MPFC GABA+ levels and rCBF in the left ventrolateral PFC (*x*, *y*, *z* = −42, 26, −12; *T* = 6.15; *Z* = 4.91; *p* = 0.011 FWE). However, Cook’s D test identified one influential data point in this association (pertaining to a UHR-T subject). Removing this subject from the analysis rendered the whole-brain correlation between MPFC GABA+ levels and rCBF in the ventrolateral PFC no longer significant at *p* < 0.05 FWE. No other whole-brain results survived FWE correction at *p* < 0.05.

### GABA+ levels and hippocampal rCBF in UHR individuals: effect of psychotic transition

**Fig. 3:**
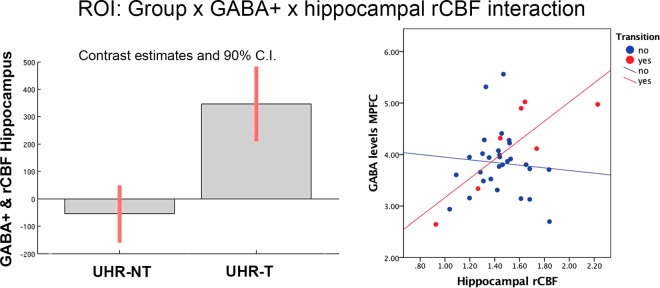
Plots depicting the group by GABA+ by rCBF interaction in the left hippocampus from ROI analysis. Regression slopes were significantly different between UHR subjects with psychotic transition (UHR-T) and UHR subjects without transition (UHR-NT). rCBF values are expressed as the ratio over global rCBF. Significant effects at *p* < 0.05 FWE

A significant group × GABA+ × rCBF interaction in the left hippocampus ROI (*x*, *y*, *z* = −18, −30, 0, *T* = 3.83, *Z* = 3.43, *p = *0.022 FWE) indicated that the strength of the association between prefrontal GABA+ levels and hippocampal rCBF in UHR subjects who went on to develop psychosis was different from that in those who did not. This reflected a strong correlation in the subgroup who developed psychosis, but the absence of a correlation in the subgroup who did not transition (Fig. 3).

Analysis of the data on self-reported anxiety as measured with the HAM-A (available in *n* = 28 subjects: 21 UHR-NT and 7 UHR-T) revealed a trend towards higher anxiety levels in UHR subjects who transitioned to psychosis than in those who did not (*t* = −2.046; *p* = 0.051). However, Pearson correlation analysis showed that HAM-A scores were not associated with GABA+ levels (UHR total: *r* = −0.160, *p* = 0.415; UHR-NT: *r* = −0.270, *p* = 0.237; UHR-T: *r* = −0.308, *p* = 0.502). Adding HAM-A scores as covariate of no interest in the rCBF group comparison rendered the whole-brain finding of hypoperfusion in a cortical midline area no longer significant (*p* = 0.093 FWE). HAM-A scores were not significantly associated with rCBF in UHR-T versus UHR-NT at *p* < 0.05 FWE (either whole-brain or with hippocampal ROI analysis), and the positive association between GABA+ levels and hippocampal rCBF remained significant after using HAM-A scores as covariate of no interest in the SPM analysis (*x*, *y*, *z* = −26, −20, 4; *T* = 3.89; *Z* = 3.33; *p* = 0.033 FWE). When antidepressant medication was used as covariate of no interest in the model the result in the hippocampus remained significant for both the across-group correlation with GABA+ levels (*x*, *y*, *z* = −28, −18, −6; *T* = 3.26; *Z* = 2.98; *p* = 0.041 FWE) and the group interaction (*x*, *y*, *z* = −18, −30, 0, *T* = 4.08, *Z* = 3.60, *p* = 0.012 FWE).

## DISCUSSION

The main finding of the present study was that MPFC GABA+ levels were related to resting hippocampal perfusion in subjects at UHR of developing psychosis. While we found no group differences in the standalone GABA ^1^H-MRS or hippocampal rCBF measures, the GABA+ correlation with hippocampal perfusion appeared to be driven by the UHR subjects who subsequently developed a psychotic disorder: there was a strong correlation in this subgroup, but no correlation in the UHR subjects who did not develop psychosis.

Preclinical models propose that cortical inhibition deficits lead to hippocampal hyperexcitability in psychosis, and that resulting increased glutamatergic outputs from the hippocampus to the striatum dysregulate subcortical dopaminergic function [49]. Recent neuroimaging studies have provided data partly consistent with such models. Resting hippocampal hyperperfusion has been described, compared with healthy controls, in patients with schizophrenia [33], and in subjects at UHR for psychosis [27, 28]. Moreover, within a UHR sample, the level of hippocampal hypermetabolism as measured using the contrast agent gadolinium to map basal cerebral blood volume has been linked to the risk of later transition to psychosis (*n* = 6) [29]. Independent work using positron emission tomography indicates that subcortical dopamine function is increased in psychosis [50] and in UHR subjects [51–54], and that the level of increase in UHR subjects is linked to the later onset of psychosis [55]. Our findings expand these data by providing evidence to suggest that distinct interactions between cortical GABA+ levels and hippocampal resting perfusion may play a role in the development of psychosis. Although we predicted that hippocampal rCBF would be correlated with MPFC GABA+ levels, we expected that the direction of the correlation would be negative rather than positive. This was based on post-mortem and preclinical evidence that PV+ interneuron expression is decreased in psychosis [1, 2, 49, 56]. Recent evidence suggests that the development of subcortical hyperdopaminergia in rodents is related to a failure of the MPFC to down-regulate medial temporal lobe activity [6], and that the MPFC can regulate hippocampal and subcortical dopamine neuron activity via the nucleus reuniens of the thalamus [57]. Human neuroimaging studies suggest that the polarity of the correlation between cortical activation and subcortical dopamine function in UHR individuals may differ depending on the cortical region involved: a positive correlation has been reported for hippocampal activation [58], but a negative correlation for prefrontal activation [59]. An additional consideration is that in preclinical and post-mortem studies the GABAergic abnormality appears to be specific to PV+ neurons [60], which account for ~40% of the cortical GABAergic interneuron population. In contrast, a limitation of ^1^H-MRS is that it quantifies total tissue concentrations as opposed to those from a particular GABA cell type, and increases could thus reflect changes in other classes of GABA interneuron. For example, compensatory mechanisms for a PV+ deficit and/or hippocampal overdrive might involve increased GABA levels in PV− interneurons [61]. An alternative explanation for the observed positive correlation between MPFC GABA+ levels and hippocampal resting perfusion is that intrinsic hippocampal GABAergic dysfunction may result in hippocampal hyperperfusion in psychosis [31] and hence GABAergic increases in MPFC [17, 21] may be compensatory in nature. Noteworthy, the measurement of hippocampal GABA function using ^1^H-MRS is technically challenging and only one such study to date has been published, reporting no significant differences between patients with schizophrenia and healthy controls [62]. Future work measuring GABAergic function in homologous regions across species with similar imaging methods may comprehensively delineate the molecular pathway linking GABAergic dysfunction to the expression of schizophrenia-like characteristics.

Exploratory analysis of the associations between levels of GABA+ in the MPFC and whole-brain rCBF revealed a significant negative association with the left ventrolateral PFC, which was strongest in the subgroup of UHR subjects who later transitioned to psychosis. Nevertheless, this effect was no longer significant once anxiety levels (HAM-A) were included in the analysis, suggesting a potential relationship between this whole-brain finding and anxiety levels in the UHR state. The ventrolateral PFC plays a major role in cognitive control processes, particularly in the cognitive regulation of emotional states [63, 64]. Difficulties with emotion regulation are proposed to be a core feature of anxiety disorders [65], in which reduced functional activation of ventrolateral PFC regions is a robust finding, along with hyperresponsivity of limbic, emotion-generation regions [66]. Although we did not have a specific hypothesis about this brain area, the direction of the association with GABA+ levels (negative) aligns with what would be hypothesized from preclinical and post-mortem findings. Although speculative, a potential explanation may be that altered GABA-perfusion interactions between cognitive control regions might lead to inefficient down-regulation of anxiety experiences in UHR subjects, particularly in those who later develop psychosis (who did show a trend towards higher self-reported anxiety than subjects who did not develop psychosis, *p* = 0.051). These findings are of interest and merit further research in larger samples.

In terms of limitations, the present study was part of a larger multimodal imaging project investigating the neurobiology of psychosis onset in UHR individuals, following an asymmetric prospective design. Both rCBF and ^1^H-MRS data could not be collected for the relatively small number of healthy controls included in the larger project, which precluded the inclusion of a comparison group in this circuit-based UHR study. Furthermore, the size of the UHR sample limited the number of subjects transitioning to psychosis by the follow-up point; the longitudinal results between UHR-T and UHR-NT must thus be interpreted with caution. Future longitudinal studies in larger UHR samples are warranted to clarify the prediction value of GABA-perfusion interactions for psychosis onset, confirm/refute the nature of our positive findings, and elucidate whether these are transdiagnostic or rather specific to different types of psychotic disorders. There was a trend towards higher levels of anxiety in the subgroup that transitioned to psychosis, but no significant association was found between anxiety scores and levels of GABA + , and the GABA-hippocampal rCBF associations and group interactions remained significant when HAM-A scores were included as covariate of no interest in the analysis. Finally, regarding MEGA-PRESS acquisition, the size of our MPFC voxel meant that some portion of anterior cingulate cortex was also included. In addition, a limitation intrinsic to all MEGA-PRESS studies is that the GABA signal contains some contribution from macromolecules, i.e. diverse proteins and lipids. However, at present, there is no evidence to suggest that the macromolecular contribution would differ between the UHT-T and UHR-NT subgroups.

In summary, our study indicates that, in individuals at ultra-high risk of developing psychosis, the level of resting hippocampal perfusion was related to prefrontal GABA+ levels. Furthermore, the data suggest that this association was present in the UHR subjects who went on to develop a psychotic disorder and absent in those who did not, although the study did not identify significant differences between UHR-NT and UHR-T subjects in either hippocampal rCBF or GABA ^1^H-MRS alone. In light of recent evidence demonstrating that peripubertal pharmacological intervention on the GABAergic system in a rodent model of psychosis can block the development of striatal hyperdopaminergia in adulthood [67–69], further research is warranted to investigate whether clinical interventions in the high-risk phase targeting this pathway may have the potential to reduce the risk of developing psychosis.

## Electronic supplementary material

Supplementary Information
