# Supplementary material for: Prefrontal GABA levels, hippocampal resting perfusion and the risk of psychosis
Source: Neuropsychopharmacology. 2018 Jan 30;43(13):2652–9. doi: 10.1038/s41386-017-0004-6 (PMC5955214; doi:10.1038/s41386-017-0004-6)
Supplement: Supplementary file 1 — Supplementary Information [file 41386_2017_4_MOESM1_ESM.docx]

**SUPPLEMENTARY INFORMATION**

**Prefrontal GABA levels, hippocampal resting cerebral blood flow and the risk of psychosis**

**Short title:** GABA, hippocampal perfusion and risk of psychosis

Gemma Modinos^1^, Fatma Şimşek ^1^, Matilda Azis^1^, Matthijs Bossong^2^, Ilaria Bonoldi^1^, Carly Samson^1^, Beverly Quinn^3^, Jesus Perez^3^, Matthew R Broome^4^, Fernando Zelaya^5^, David J Lythgoe^5^, Oliver Howes^1^, James M Stone^5^, Anthony A Grace^6^, Paul Allen^1,7^, Philip McGuire^1^

^1^ Department of Psychosis Studies, Institute of Psychiatry, Psychology & Neuroscience, King’s College London, UK

^2^ Department of Psychiatry, Brain Center Rudolf Magnus, University Medical Center Utrecht, Netherlands

^3^ CAMEO Early Intervention in Psychosis Service, Cambridgeshire and Peterborough NHS Foundation Trust, Cambridge, UK; Department of Psychiatry, University of Cambridge, Cambridge, UK; Department of Neuroscience, Instituto de Investigacion Biomedica de Salamanca (IBSAL), University of Salamanca, Spain

^4^ Department of Psychiatry, University of Oxford, Oxford, UK; ; Oxford Health NHS Foundation Trust, Oxford, UK

^5^ Department of Neuroimaging, Institute of Psychiatry, Psychology & Neuroscience, King’s College London, UK

^6^ Department of Neuroscience, University of Pittsburgh, PA, USA

^7^ Department of Psychology, University of Roehampton, UK

**Corresponding author**: Gemma Modinos, Department of Psychosis Studies, Institute of Psychiatry, Psychology & Neuroscience, King’s College London, 16 De Crespigny Park, SE5 8AF, London, UK. Email: [gemma.modinos@kcl.ac.uk](mailto:gemma.modinos@kcl.ac.uk)

**SUPPLEMENTARY RESULTS**

**rCBF in UHR Individuals: Relationship to Clinical Outcome**

**
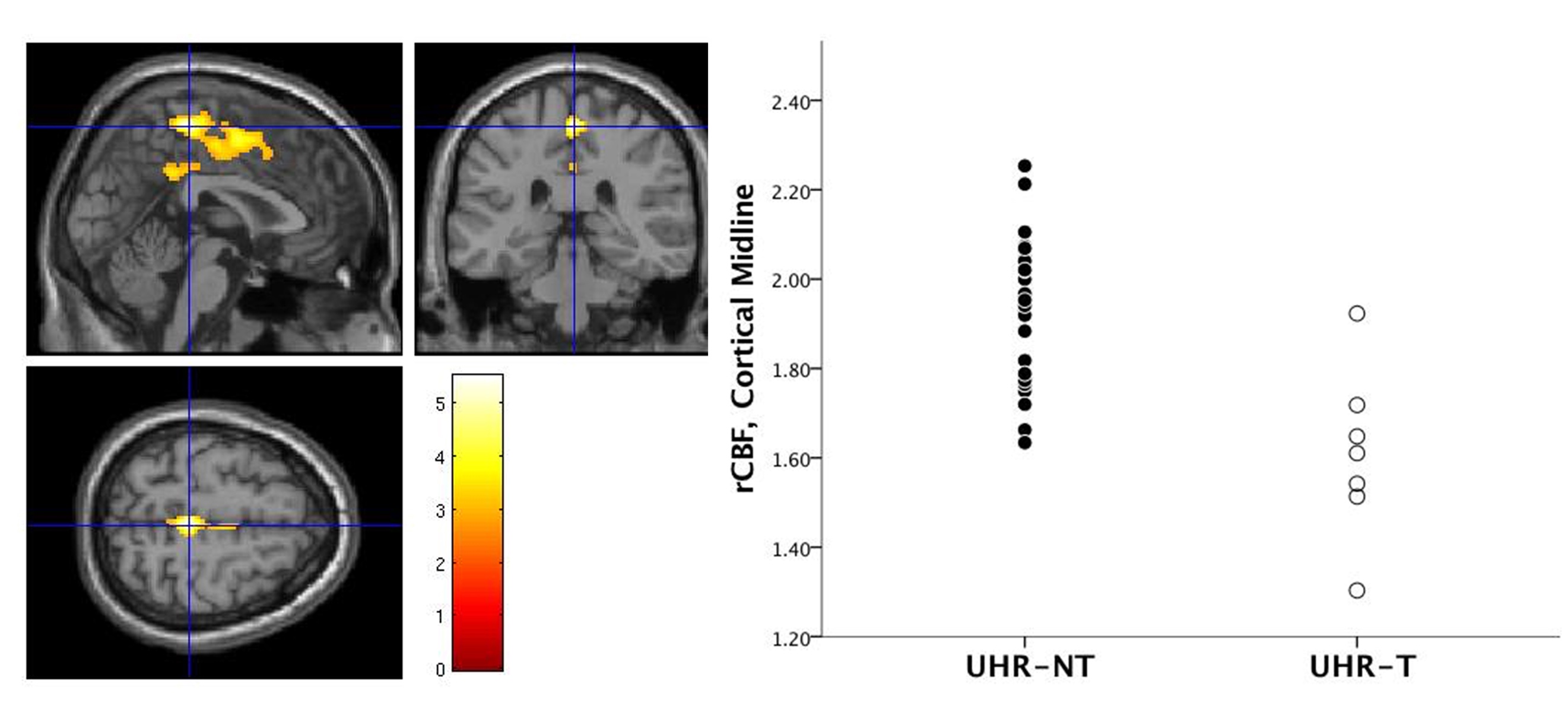
**

**Figure S1.** Section overlay and plot depicting rCBF differences between the UHR-NT and UHR-T groups at the whole-brain level. rCBF values are expressed as ratio over global rCBF. Significant effects at *p* < 0.05 FWE, shown at *p* < .005 uncorrected for display purposes.
